# A semi-supervised approach using label propagation to support citation screening

**DOI:** 10.1016/j.jbi.2017.06.018

**Published:** 2017-08

**Authors:** Georgios Kontonatsios, Austin J. Brockmeier, Piotr Przybyła, John McNaught, Tingting Mu, John Y. Goulermas, Sophia Ananiadou

**Affiliations:** a National Centre for Text Mining, School of Computer Science, University of Manchester, Manchester, United Kingdom; b School of Electrical Engineering, Electronics and Computer Science, University of Liverpool, Liverpool, United Kingdom

**Keywords:** Active learning, Label propagation, Citation screening, Semi-supervised learning, Text classification

## Abstract

Citation screening, an integral process within systematic reviews that identifies citations relevant to the underlying research question, is a time-consuming and resource-intensive task. During the screening task, analysts manually assign a label to each citation, to designate whether a citation is eligible for inclusion in the review. Recently, several studies have explored the use of active learning in text classification to reduce the human workload involved in the screening task. However, existing approaches require a significant amount of manually labelled citations for the text classification to achieve a robust performance. In this paper, we propose a semi-supervised method that identifies relevant citations as early as possible in the screening process by exploiting the pairwise similarities between labelled and unlabelled citations to improve the classification performance without additional manual labelling effort. Our approach is based on the hypothesis that similar citations share the same label (e.g., if one citation should be included, then other similar citations should be included also). To calculate the similarity between labelled and unlabelled citations we investigate two different feature spaces, namely a bag-of-words and a spectral embedding based on the bag-of-words. The semi-supervised method propagates the classification codes of manually labelled citations to neighbouring unlabelled citations in the feature space. The automatically labelled citations are combined with the manually labelled citations to form an augmented training set. For evaluation purposes, we apply our method to reviews from clinical and public health. The results show that our semi-supervised method with label propagation achieves statistically significant improvements over two state-of-the-art active learning approaches across both clinical and public health reviews.

## Highlights

• Systematic reviews can benefit from automatically screening relevant citations.
• We propose a new method that improves the performance by using similar citations.
• We utilise unlabelled documents by propagating labels in close neighbourhood.
• A space of spectral embeddings is used for better distance representation.
• Results on clinical and biomedical domains show consistent improvements.

## Graphical abstract

**Figure:**
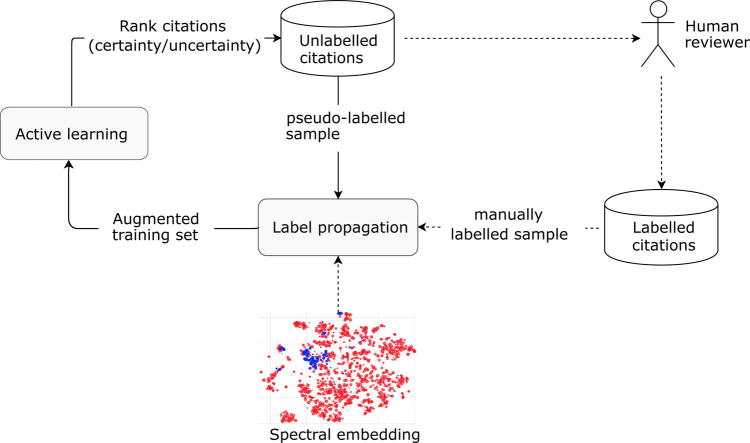

## 1 Introduction

Systematic reviews are used to identify relevant citations and answer research questions by gathering, filtering, and synthesising research evidence. A primary objective of any systematic review is to minimise publication bias [1] by analysing all citations relevant to the review. To identify and subsequently analyse every possible eligible study, reviewers need to exhaustively filter out citations (retrieved by searches to literature databases) that do not fulfill the underlying eligibility criteria. Developing systematic reviews is a time-consuming and resource intensive process that can take more than a year, with up to half of this time being spent searching and screening hits. As an example, an experienced reviewer requires 30 s on average to decide whether a single citation is eligible for inclusion in the review, although this can extend to several minutes for complex topics [2]. This amounts to a considerable human workload, given that a typical screening task involves manually screening thousands of citations [3], [4], [5].

To reduce the time and cost needed to complete the screening phase of a systematic review, researchers have explored various techniques, including crowdsourcing and text mining methods. Crowdsourcing approaches efficiently address tedious tasks, e.g., assessing the quality of Wikipedia articles [6], by re-distributing the overall workload to a large network of people. In the context of systematic reviews, the EMBASE screening project,^1^ a Cochrane initiative, adopts a crowdsourcing approach to identify reports of randomised controlled trials (RCTs) and quasi-RCTs in the EMBASE bibliographic database. Two years after the project started, 4606 crowd workers have processed a total number of 1 million EMBASE abstracts. Regarding the quality of the screening decisions, the crowd workers were found to be very accurate achieving a sensitivity and specificity performance of 99%.

In addition to crowdsourcing approaches, previous studies have investigated the use of automatic text classification to facilitate citation screening of systematic reviews [5], [7]. In citation screening supported by automatic text classification, a human reviewer needs to screen only a subset of the retrieved citations. The process starts with a subset of citations manually annotated with labels, which denote whether the citation should be included or excluded. The citations paired with the labels serve as the training examples for the automatic classifier. In a supervised learning manner, the classifier is then trained on the manually annotated set to learn how to discriminate between relevant and irrelevant citations. As a final step, the trained classifier is applied to automatically screen the remaining unlabelled citations.

In this paper, we focus on a special case of automatic text classification known as feedback-based or active learning classification [2], [8], [9], [10], [11]. Active learning classification approaches are supervised machine learning methods that are iteratively trained on an increasing number of manually labelled citations. At each learning cycle, the model selects a small sample of citations and interactively requests a human analyst to manually label the citations. The manually labelled sample of citations is added to the training set and the model is retrained (updated). Results obtained by previous work [2], [8] demonstrate that active learning classification approaches can substantially decrease the screening workload without reducing the sensitivity of the review (i.e., the method identifies 95–100% of relevant citations). However, a drawback of existing active learning methods is that the underlying model yields a low performance during the early learning iterations due to the limited number of labelled citations used as training instances. This can be explained because active learning methods exploit machine learning models whose hypothesis space, i.e., the possible set of decision boundaries, is constrained by the number training instances. Thus, a small number of training samples in the initial stages may result in poor classification performance [12].

Previous work [5], [13] has outlined that the early identification of eligible citations presents several advantages to systematic reviewers and can significantly accelerate the overall citation screening process. As an example, O’Mara-Eves et al. [5] argued that, in a manually conducted citation screening task, reviewers tend to screen at a lower rate during the initial stages of the task while they incrementally increase their screening rate only after processing a larger number of eligible citations. Thus, the prioritisation of eligible citations during the initial active learning iterations can enable reviewers to establish a higher screening rate early in the process, reducing in this way the overall time needed to complete the citation screening task.

Based upon this, we propose a semi-supervised active learning method to improve the classification performance of active learning during the early stages of screening. In our approach, we adopt the ‘cluster assumption’ [14], which states that instances that are similar to each other will often share the same label. Accordingly, we use label propagation [15] to copy the label from a manually labelled citation to similar unlabelled citations (which are nearby in the feature space). These pseudo-labelled samples are used as additional training data for the classifier. To compute pairwise similarities between labelled and unlabelled instances, we explore two different feature representations of citations: (a) a bag-of-words feature space which consists of words that occur in the title and/or in the abstract of the citation and (b) a spectral embedding space that approximates the similarities of the bag-of-words representation based on their relative location in a lower dimensional space (neighbouring instances in the embedding should share similar content).

The label propagation step, which extends the training set with additional pseudo-labelled instances, can be used with any active learning method. Here, we integrate the proposed label propagation method with two existing active learning strategies, namely a certainty-based [8] and an uncertainty-based active learner [2]. The two strategies have different motivations. Uncertainty-based sampling [16], [11] learns to discriminate between eligible and ineligible citations by requesting feedback from an analyst on citations that are more likely to change the current model. Certainty-based sampling [8], [17] seeks to identify the relevant citations as early as possible, which is a useful strategy for systematic reviews [5].

For experimentation, we investigate the performance of the semi-supervised active learning method when applied to both clinical and public health systematic reviews. Such reviews are becoming increasingly difficult to manually develop and update due to the exponential growth of the biomedical literature (e.g., on average 75 trials and 11 systematic reviews are published daily in MEDLINE [18]). As an example, only a third of systematic reviews in the Cochrane library are being frequently updated with new relevant evidence published in the literature [19]. Thus, semi-automatic methods that can potentially accelerate the development of clinical and public health reviews are needed [20].

The contributions that we make in this paper can be summarised in the following points: (a) we propose a new semi-supervised active learning method to facilitate citation screening in clinical and public health reviews; (b) we show that a low-dimensional spectral embedded feature space can more efficiently address the high terminological variation in public health reviews versus the bag-of-words representation; and (c) experiments across two clinical and four public health reviews demonstrate that our method achieves significant improvements over two existing state-of-the-art active learning methods when a limited number of labelled instances is available for training.

### 1.1 Previous work on automatic citation screening

Previous approaches to automatic citation screening can be coarsely classified into automatic text classification and active learning classification methods. Aphinyanaphongs and Aliferis [21] proposed one of the earliest automatic text classification approaches for identifying high-quality and content-specific research articles useful for evidence-based reviews. They experimented with different supervised machine learning methods including a naïve Bayes classifier [22], boosting [23] and a support vector machine (SVM) [24]. As the feature representation for articles, they exploited words occurring in the title and/or in the abstract, the publication type (e.g., randomised control trial) and MeSH terms. Experimental results determined that the SVM classifier achieved an improved classification performance over the naïve Bayes and boosting classifiers.

Cohen et al. [13] applied an automatic text classification model in 15 systematic reviews relating to drug class efficacy for disease treatment. They used a modified version of the voted perceptron algorithm [25], i.e., a maximal-margin classifier which, similarly to an SVM, tries to find a hyperplane to separate relevant from irrelevant citations. As in previous work [21], they used a bag-of-words feature representation complemented by publication type and MeSH term features. In order to better address the high-recall requirement of systematic reviews—that is, reviewers need to identify all relevant citations for inclusion in the review—they introduced a bias weight to control the learning rate of positive (relevant) and negative (irrelevant) instances. Their results demonstrated a significant reduction in the screening workload in 11 out of the 15 reviews. Matwin et al. [26] explored the use of a factorised version of the naïve Bayes classifier as opposed to the voted perceptron method used in [13]. The authors argued that automatic screening decisions obtained by Bayesian classification approaches are easier to interpret than screening decisions derived by pattern recognition tools such as the voted perceptron classifier or SVMs. In addition to this, they defined feature weights to assign a higher priority to publication type and MeSH terms than to bag-of-words features. The naïve Bayes screening model was shown to achieve a better classification performance than the voted perceptron classifier on the 15 drug-specific systematic reviews.

Frunza et al. [27] employed an ensemble classification method consisting of multiple naïve Bayes models. Each model was trained to predict a different inclusion criterion of the underlying review (e.g., inclusion of primary citations). Individual screening decisions were then combined into a voting scheme to classify citations as being eligible or ineligible for the review. They experimented with a large scale medical review containing more than 47,000 citations to be screened. Results determined that the ensemble classification method substantially outperformed a monolithic naïve Bayes classifier trained only on global screening decisions. Howard et al. [28] developed a regularised log-linear classification model which exploits two types of features, namely bag-of-words weighted by TF-IDF and topic-based features extracted by Latent Dirichlet Allocation (LDA) [29]. Experiments across 20 systematic reviews were performed, demonstrating a robust precision and a very high recall of 95%. Elsewhere, García Adeva et al. [30] studied the contribution of different segments of a citation (e.g., title, abstract or both) to the overall classification performance; Shemilt et al. [31] showed that SVM-based text classifiers can significantly reduce the screening workload of very large scoping reviews; while Timsina et al. [32] investigated different strategies to mitigate the class imbalance between eligible and ineligible citations which is known to affect the performance of the classification model.

One limitation of automatic classification approaches is that the underlying models rely upon fine tuning of weighting parameters to achieve high recall. Cohen et al. [13] noted that the value of the weighting parameter that results in an acceptable recall performance (i.e., ⩾95%), “*varies greatly*” across the 15 drug-specific systematic reviews. Moreover, the authors reported that in one out of the 15 reviews the model was unable to converge to high recall levels for any value of the parameter. This observation was subsequently confirmed by Bekhuis and Demner-Fushman [33]. In their study, they evaluated different automatic classification methods, including naïve Bayes, SVM, and k-nearest neighbour, and showed that the models achieve low recall when using default parameter settings. To improve the recall of automatic text classification, they employed a grid optimisation technique that identifies optimal parameter settings for the machine learning models.

A second-generation group of techniques, including our approach, explores the use of active learning to train text classification models. Unlike automatic classification methods that train machine learning models on predefined training sets, i.e., randomly drawn samples of the full set, active learning models start with a very small random set and then incrementally select samples to be manually labelled and added to the training set. Wallace et al. [2] presented an active learning strategy based on SVMs for citation screening. Their method uses uncertainty sampling to select instances lying closer to the classification hyperplane (i.e., the decision threshold between relevant and non-relevant citations) for inclusion in the training set. Uncertainty sampling assumes that low confidence instances can be used to train a machine learning model more efficiently (by refining the classification hyperplane) and thus to improve the performance of the active learner. The authors reported that the uncertainty-based active learning method was able to reduce the number of items that needed to be manually screened by 40–50% in clinical systematic reviews.

Miwa et al. [8] employed an SVM-based active learner with certainty-based sampling that selects high confidence instances to be included in the next training cycles as opposed to uncertainty sampling [2]. Certainty-based sampling is known to better address the skewed distribution of positive and negative instances that is likely to occur in systematic reviews [17]. In addition to certainty sampling, a weighting method was used to assign a higher importance to relevant instances. The weighting method was shown to further alleviate class imbalance. Experimental results determined that active learning with certainty sampling and weighting compares favourably to active learning with uncertainty sampling across clinical and public health reviews.

Previous work has obtained impressive results using active learning classification methods for citation screening. However, existing active learning methods require a large amount of labelled data to yield a robust performance. In this study, we propose a novel semi-supervised active learning method that is able to learn from both labelled and unlabelled data. A similar approach was recently presented in Liu et al. [34], comparing existing semi-supervised classifiers [35], [15], [36] to an SVM-based automatic classification method. Although promising results were reported, that study failed to demonstrate the potential benefits from using semi-supervision within active learning. In our experiments, we demonstrate that semi-supervision improves upon the performance of both certainty and uncertainty-based active learning when a limited number of manually annotated instances is available for training.

## 2 Methods

In this section, we present the overall architecture of our semi-supervised active learning method. We then provide implementation details of the label propagation and the spectral embedding feature space that we use to efficiently transfer classification labels from manually labelled to unlabelled instances.

### 2.1 Semi-supervised active learning for citation screening

**Fig. 1:**
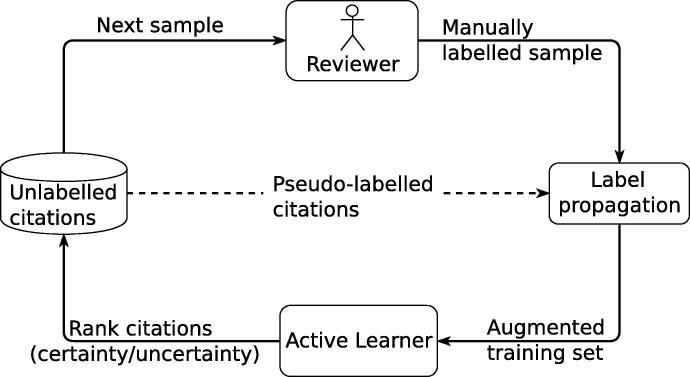
Architecture of the semi-supervised active learning approach used for citations screening.

Fig. 1 shows the overall architecture of the proposed semi-supervised active learning method. The process starts with a pool of unlabelled citations. In the first iteration, a human reviewer manually labels a small, randomly selected sample of citations. The label propagation method generates additional training instances by copying the class labels of previously labelled instances to unlabelled instances most similar in the feature space. These automatically labelled instances are combined with the manually labelled instances to form an augmented training set, which is used for training the text classification model. In the final step of the active learning cycle, the active learning model is applied to the pool of remaining unlabelled instances, the label of each instance is predicted, along with a measure of certainty for the label, and the reviewer re-initiates the active learning cycle by annotating the highest ranked citations in the unlabelled set, where the ranking depends on whether the user is interested in performing certainty- or uncertainty-based active learning. For certainty-based sampling, the highest ranked instances are those most likely to be positive (eligible) according to the current model, while uncertainty-based sampling prioritises instances with the lowest confidence of the classifier’s prediction.

The semi-automatic citation screening will terminate when a stopping criterion is met. In this work, we allow the model to annotate the complete set of unlabelled citations and report the performance on various intermediate iterations of the active learning method. In scenarios where the semi-supervised active learning method is applied to ongoing systematic reviews, the reviewing team can stop the screening process when the majority of relevant citations has been identified. For example in the clinical domain, active learning approaches converge to recall levels of 95–100% after processing 50–60% of the total number of articles [2], [8].

### 2.2 Label propagation

Our semi-supervised method automatically propagates the class label from a labelled instance to the neighbouring unlabelled instances. Formally, given a manually annotated instance (d,y), where d∈{1,…,n} enumerates the citations, *n* is the total number of citations, and y∈{0,1} is the corresponding class label where 0 indicates a citation should be excluded and 1 designates inclusion, our goal is to determine a set $N_{d}\subset \{1\text{,}\ldots \text{,}n\}⧹d$ of the *k*-nearest neighbours to *d*.

The class label of *y* is then assigned to any neighbours that are unlabelled and these neighbours are used as additional training instances for the classifier. If an unlabelled instance is the neighbour of multiple labelled samples, the label of the closest instance is assigned.

To calculate the distance between two instances, *d* and $d^{\prime }$, we use the cosine of the angle between the vector representations of *d* and $d^{\prime }$:

(1) $$d(v_{d}\text{,}v_{d^{\prime }})=1-\cos\theta =1-\frac{\langle v_{d}\text{,}v_{d^{\prime }}\rangle }{\left\|v_{d}\|\;\|v_{d^{\prime }}\right\|}$$

where $\langle v\text{,}u\rangle =\sum_{i=1}^{q}v_{i}u_{i}$ indicates the inner-product (dot-product) between vectors $v\text{,}u\in R^{q}\text{,}\;q$ is the dimension of the vector space and $\|v\|=\sqrt{\sum_{i=1}^{q}v_{i}^{2}}$ is the Euclidean norm. With the normalisation, the cosine similarity provides a fair comparison between vectors of different magnitude, which is especially important for the bag-of-words representation where the magnitude is proportional to the number of words. We explore two different vector representations of citations formed from the title and abstract text: (a) bag-of-words representation and (b) spectral embedded feature space, a lower dimensional embedding of the bag-of-words space. The spectral embedded feature space has been shown to better capture cluster structures of instances [14]; thus, the distance between instances of the same label is expected to be smaller in the spectral space.

To better illustrate the underlying ‘cluster assumption’ we conducted two experiments. Firstly, we computed the distribution of distances in the spectral embedded space between citations that share the same class label and between citations with contrasting labels. Secondly, we use t-SNE [37], a visualisation algorithm for high-dimensional data that maps the spectral embedded representation of citations into a two-dimensional space. The t-SNE algorithm is able to preserve some of the topology of the high-dimensional data in the lower dimensional space (i.e., adjacent points in the visualisation are also close to each other in the original space).

**Fig. 2:**
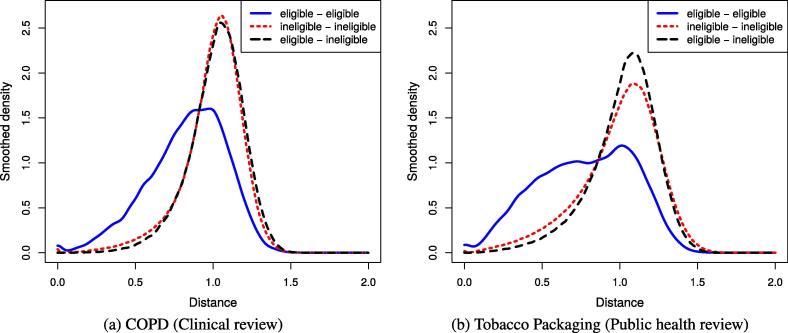
Smoothed density function of the distances between pairs of citations in a spectral embedded feature space, which shows that distances between two eligible citations is typically less than the distance between arbitrary pairs of citations.

Fig. 2, shows the smooth density functions of the empirical distribution of distances between pairs of citations in a clinical review (COPD) and in a public health review (Tobacco Packaging), respectively. In both datasets, we observe that pairs of eligible citations have relatively small distances, followed by pairs of ineligible citations, while mixed pairs (eligible-ineligible) present the highest mean distance. Small distances between pairs of eligible citations justify propagating labels between neighbouring citations.

**Fig. 3:**
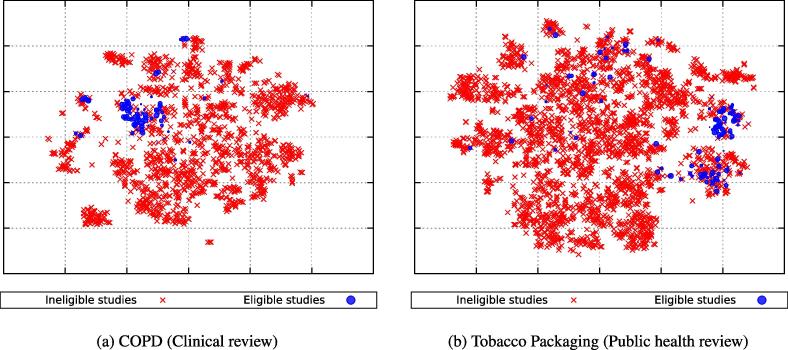
t-SNE visualisation of citations, encoded in a spectral embedded feature space, of a clinical and a public health review. Solid blue dots indicate eligible citations and red crosses indicate ineligible citations. (For interpretation of the references to colour in this figure legend, the reader is referred to the web version of this article.)

Fig. 3 shows a t-SNE visualisation of eligible and ineligible citations. With respect to the clinical review (COPD), we observe that citations tend to be organised into homogeneous clusters where instances of the same class label are close to each other. In the case of the public health review (Tobacco Packaging), we note similar cluster structures, although several eligible instances are scattered across the space. The apparent singleton clustering of eligible citations can be explained by the fact that public health reviews often cover complex, multi-disciplinary topics (e.g., social sciences, psychology, economics) [8], [5]. The isolated but relevant citations remain a challenge to identify using automatic text classification methods.

#### 2.2.1 Spectral embeddings

The neighbourhoods used in the label propagation depend on the choice of vector representation for the citations. One option is the ubiquitous bag-of-words representation, where citations are deemed to be close if they share many of the same words. The dimensionality of the bag-of-words representation is controlled by the size of the vocabulary, and two different words are always treated as orthogonal dimensions (even if they are semantically similar). Because of the potential large variations in vocabulary between different citations, this representation is not always optimal.

As an alternative, we use a spectral embedding technique [14], which is a data-dependent representation that can preserve semantic similarity within a lower dimensional space. In particular, a lower-dimensional embedding is akin to principal component analysis (PCA) in that it will preserve the dominant patterns of similarity, thereby alleviating noise related to uncommon words and variation. Other alternatives to bag-of-words representations based on generative topic models, namely LDA, or distributed vector representations of portions of texts trained via shallow neural networks [38], have also been shown to improve the simulated performance of active learning on systematic review datasets [8], [39]. The advantage of spectral embeddings over these methods is mainly computational, in that it can be computed very quickly using standard linear algebra libraries. A comparison of the effect of the representation choice on the semi-supervised active learning is left for future research.

We compute a spectral embedded representation based on the eigendecomposition of the normalised similarity matrix between pairs of instances [14], [40]. Let *X* denote the TF-IDF bag-of-words feature matrix where $X_{d\text{,}w}=c_{d\text{,}w}\log\frac{1}{f_{w}}$ is the product of the term-count $c_{d\text{,}w}$ for word *w* in citation *d*, and $f_{w}$ is the fraction of the citations that contain *w* for w∈{1,…,m}, where *m* is the number of words in the vocabulary, and d∈{1,…,n}. From its definition, *X* is a non-negative matrix since $c_{d\text{,}w}$ and $\log\frac{1}{f_{w}}$ are always non-negative. We use a normalised representation the vector for each citation stored in the matrix $R={\left[r_{1}\text{,}r_{2}\text{,}\ldots \text{,}r_{n}\right]}^{T}$ where $R_{d\text{,}w}=\sqrt{X_{d\text{,}w}}/\sqrt{\sum_{w^{\prime }}X_{d\text{,}w^{\prime }}}$. Due to the above mentioned positivity and normalised representation, the inner product between two normalised vectors $r_{d}$ and $r_{d^{\prime }}$ yields the Bhattacharya coefficient measure [41] of similarity $C_{d\text{,}d^{\prime }}=\langle r_{d}\text{,}r_{d^{\prime }}\rangle$ for $d\text{,}d^{\prime }\in \{1\text{,}\ldots \text{,}n\}$, where $0⩽C_{d\text{,}d^{\prime }}⩽1$. The n×n matrix *C* is positive semidefinite (it has no negative eigenvalues) and its diagonal entries are 1. A truncated eigendecomposition of *C* can be used to form an embedding. However, this decomposition tends to represent only the largest groups of highly similar instances, while the remaining instances remain near the origin of the embedding coordinate system. To address this problem, we use a symmetrically normalised version of *C*. This normalisation approach has been theoretically justified and popularised for spectral clustering [40] and embedding [14]. The symmetrically normalised matrix is computed as $\overset{\sim }{C}=D^{-1/2}{\mathrm{CD}}^{-1/2}$ where *D* is a diagonal matrix with entries $D_{d\text{,}d}=\sum_{d^{\prime }}C_{d\text{,}d^{\prime }}$ for d∈{1,…,n}. Based on the symmetrically normalised matrix $\overset{\sim }{C}$, we compute an eigendecomposition $U\Lambda U^{T}=\overset{\sim }{C}$ where *U* is a unitary matrix with the eigenvectors as columns and Λ a diagonal matrix with the eigenvalues on the diagonal. The diagonal elements can be sorted such that $\lambda_{1}⩾\cdots ⩾\lambda_{p}$ are the first *p* entries corresponding to the largest eigenvalues. We then compute an embedding of dimension *p* according to:

(2) $$Z=[u_{1}\sqrt{\lambda_{1}}\text{,}\ldots \text{,}u_{p}\sqrt{\lambda_{p}}]={\left[z_{1}\text{,}z_{2}\text{,}\ldots \text{,}z_{n}\right]}^{T}\in R^{n\times p}$$

where $u_{1}\text{,}\ldots \text{,}u_{p}$ are eigenvectors associated with the *p* largest eigenvalues. In this notation, $z_{d}$ is the $d^{\mathrm{th}}$ row of *Z* and is the embedding coordinates for the $d^{\mathrm{th}}$ instance. In our experiments, we set the dimensionality of the embedded feature space to p=50.

In general, $\overset{\sim }{C}$ is a non-sparse matrix. Hence, computing even a truncated eigendecomposition of $\overset{\sim }{C}$ becomes computationally expensive for a large number of instances. However, $\overset{\sim }{C}$ can be implicitly defined in terms of the sparse normalised TF-IDF matrix *R* and the diagonal matrix $D:\overset{\sim }{C}=D^{-1/2}{\mathrm{RR}}^{T}D^{-1/2}$. In this form, the matrix-vector multiplications required to obtain the eigendecomposition can be efficiently computed as $\overset{\sim }{C}x=D^{-1/2}{\mathrm{RR}}^{T}D^{-1/2}x=D^{-1/2}(R(R^{T}(D^{-1/2}x)))$, since this equation consists of a series of sparse matrix-vector multiplications.

## 3 Results

In this section, we present experiments to evaluate the proposed semi-supervised active learning methods. Firstly, we describe the 6 systematic review datasets which we used in our experiments. Secondly, we define the evaluation metrics for assessing the citation screening methods. Finally, we compare our method against two existing active learning approaches across the 6 evaluation datasets.

### 3.1 Data

Table 1 summarises various characteristics of the employed systematic review datasets, including the underlying scientific domain (clinical or public health domain), the number of instances and the ratio of eligible to ineligible (positive to negative) instances. We use two systematic reviews from the clinical domain (COPD and Proton Beam) and four reviews from the public health domain (Cooking Skills, Sanitation, Tobacco Packaging and Youth Development). The clinical reviews are publicly available datasets and were previously used by Wallace et al. [2] to evaluate an uncertainty-based active learner. The public health reviews were developed by the EPPI-Centre^2^ and reused by Miwa et al. [8] to investigate the performance of both certainty and uncertainty-based active learners.

**Table 1:** Characteristics of the employed systematic review datasets.

	Domain	# Instances	# eligible / # ineligible
Proton Beam	Clinical	4751	0.05
COPD	Clinical	1606	0.14
Cooking Skills	Public health	11,515	0.02
Sanitation	Public health	5464	0.10
Tobacco Packaging	Public health	3210	0.05
Youth Development	Public health	15,544	0.11

With regard to the size of the employed datasets, the Youth Development review is the largest systematic review consisting of 15,544 abstracts to be screened. On the assumption that a human reviewer screens an average of one abstract in 30 s, manually screening the entire Youth Development dataset requires approximately 130 h of work; this is over 3 weeks at 40 h per week. Moreover, it should be noted that both the clinical and the public health datasets are highly imbalanced, containing far fewer eligible than ineligible citations. Such imbalanced datasets constitute challenging cases for machine learning methods [2], [8], [13], [31].

### 3.2 Evaluation settings

We have evaluated six automatic screening methods: active learning with certainty sampling (AL-C) [8]; active learning with uncertainty sampling (AL-U) [2]; two semi-supervised active learning models that propagate classification labels using a bag-of-words feature space (i.e., SemiBoW-AL-C for certainty sampling and SemiBoW-AL-U for uncertainty sampling); and two semi-supervised active learning methods that use a spectral embedded space for label propagation (SemiSpectral-AL-C and SemiSpectral-AL-U). The semi-supervised models (SemiBoW-AL and SemiSpectral-AL) correspond to our novel methods (with the number of neighbours for label propagation fixed^3^ at k=3), while AL-C and AL-U are used as baseline methods. All methods use linear SVMs.

As evaluation metrics, we use yield and burden [8], [42], [2]: yield is the fraction of relevant citations identified by a given automatic screening method, and burden is the fraction of the total number of citations that a human reviewer needs to manually screen. They are mathematically defined as:

(3) $$\mathrm{yield}=\frac{{\mathrm{tp}}^{h}+{\mathrm{tp}}^{a}}{{\mathrm{tp}}^{h}+{\mathrm{tp}}^{a}+{\mathrm{fn}}^{a}}$$

(4) $$\mathrm{burden}=\frac{n^{h}+{\mathrm{tp}}^{a}+{\mathrm{fp}}^{a}}{n}$$

where tp,fp,fn,n denote the number of true positives, false positives, false negatives, and total number of instances; and the superscripts $\cdot^{h}$ and $\cdot^{a}$ denote human and automatic labelling, respectively. We assume manual labelling is correct, such that ${\mathrm{tp}}^{h}+{\mathrm{tn}}^{h}=n^{h}$ where $n^{h}+n^{a}=n$. The goal of an active learning citation screening method is to maximise yield (proportion of examined citations that are eligible) while minimising burden (human workload involved in the screening phase). At 100% burden, a (human) systematic reviewer has screened the complete citation list and all eligible citations are identified (100% yield).

In order to provide a single evaluation metric of the active learning performance, we use utility that considers both yield and burden. Utility is computed as follows:

(5) $$\mathrm{utility}=\frac{\beta \times \mathrm{yield}+(1-\mathrm{burden})}{\beta +1}$$

where β is a weighting factor used to determine the importance of yield and burden. Given that the identification and inclusion of all relevant citations is a critical feature of each systematic review, a high value of yield becomes more important than a low value of burden [5]. Wallace et al. [43] noted that, according to experts, yield is 19 times more important than burden. Based upon this, we set the weighting factor as β=19.

For assessment, the values of yield, burden and utility are computed for each round of active learning-based screening according to the predictions made by the classifier. These predictions cover the remainder of the unlabelled instances, including those that will not be presented to the human reviewer. That is, these metrics quantify the performance as if the active learning process terminated and the human reviewer annotated the instances predicted to be relevant. In the case of the certainty-based screening, the instances presented to the human reviewer are those most likely to be relevant, while for uncertainty-based screening, the subset presented to the reviewer may be a mixture of relevant and irrelevant instances for which the classifier has the least confidence.

We use the *average utility* to quantify the expected utility when stopping earlier than a specified number of manually labelled instances. The average utility performance after *R* iterations of active learning is computed as

(6) $$\mathrm{average}\;\mathrm{utility}@R=\frac{1}{R}\overset{R}{\underset{r=1}{\sum }}{\mathrm{utility}}_{r}$$

where ${\mathrm{utility}}_{1}$ is the utility performance of the first active learning iteration and ${\mathrm{utility}}_{r}$ is the utility of the *r*-*th* iteration. The advantage of average utility is that it considers the utility performance across previous iterations, providing a smoother metric for evaluation purposes.

### 3.3 Overview of results

We evaluate the utility performance of certainty-based (AL-C, SemiBoW-AL-C, SemiSpectral-AL-C) and uncertainty-based (AL-U, SemiBoW-AL-U, SemiSpectral-AL-U) active learning models when applied to one clinical and one public health review, i.e., Proton Beam and Tobacco Packaging, respectively (please refer to the supplementary material for the performance of the models on the other datasets). Additionally, we record the performance of a conventional, manually conducted citation screening process (i.e., *Manual*). The performance of active learning depends upon the initial seed of manually labelled instances, which are randomly selected. Based upon this, we repeat each citation screening experiment 10 times and we report the utility averaged across the 10 runs. The standard deviation of utility values over the 6 systematic review datasets is also recorded.

**Fig. 4:**
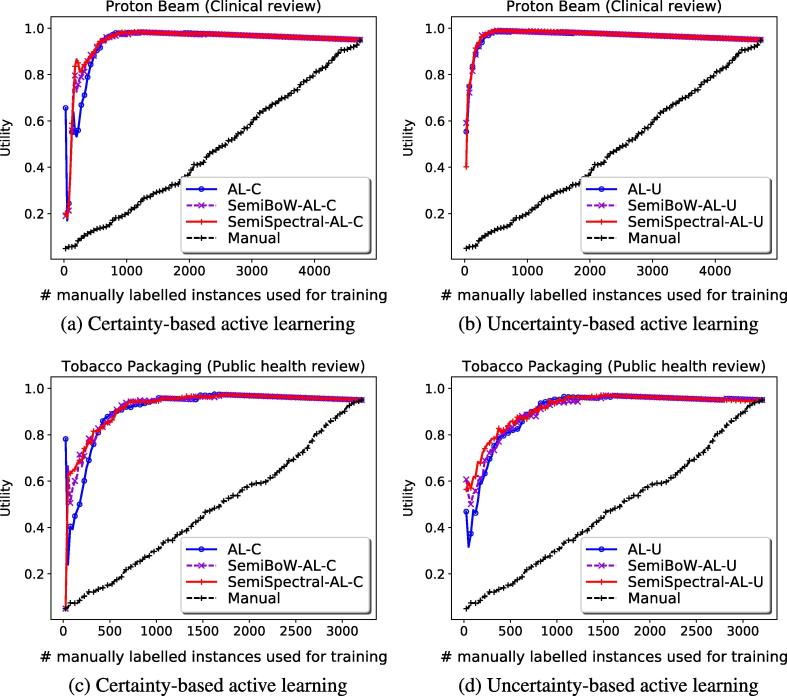
Utility performance achieved by certainty and uncertainty-based active learning models when applied to a clinical (Proton Beam) and a public health (Tobacco Packaging) review.

Fig. 4a compares the utility performance of certainty-based models on the clinical Proton Beam dataset. It can be noted that during the initial learning iterations, the proposed semi-supervised active learning models (i.e., SemiBoW-AL-C and SemiSpectral-AL-C) achieve improved utility compared to the baseline active learning method (i.e., AL-C). Specifically, the SemiSpectral-AL-C method shows superior utility when 5% of the citations is manually labelled and used for training (+9% utility over the SemiBoW-AL-C method and +30% over the baseline AL-C method). Moreover, all three active learning models obtain substantial improvements over the manual screening process (the utility of manual screening increases approximately linearly with the number of manually labelled instances). This demonstrates the effectiveness of active learning citation screening over conventional screening approaches. By comparing the uncertainty-based methods on the same clinical dataset (see Fig. 4b), we observe that the semi-supervised models (SemiBoW-AL-U and SemiSpectral-Al-U), shows marginal performance improvements over the baseline AL-U method (the SemiSpectral-Al-U improves the utility by only 1% over the baseline when 5% of the citations is used for training). This can be explained by the fact that the baseline method quickly converges to achieve very high utility when few labelled instances are available for training (e.g., 92% utility performance using 5% of the citations for training).

Fig. 4c and d shows the utility of certainty-based and uncertainty-based models, respectively, when applied to a public health review (i.e., Tobacco Packaging). With regard to the certainty-based active learning methods (see Fig. 4c), we observe that the utility achieved by the two semi-supervised methods (i.e., SemiBoW-AL-C and SemiSpectral-AL-C) increases more rapidly than the performance obtained by the AL-C baseline method when few labelled instances are available for training. Performance gains over the baseline method range between 15% and 20% when 5% of the instances are manually labelled and used for training and between 1% and 5% when 10% of the instances are used for training. Fig. 4d illustrates the performance of the uncertainty-based models when applied to the public health Tobacco Packaging review. Here, we note that the semi-supervised method that exploits a spectral embedded feature space for label propagation (i.e., SemiSpectral-AL-U) obtains the best utility during the initial active learning iterations (i.e., +10% over the SemiBoW-AL-U method and +17% over the baseline AL-U method when 5% of citations is used for training). In subsequent learning iterations (i.e., *>*10% of the citations is manually screened), the models converge to approximately the same yield and burden.

Table 2 summarises the average utility of certainty-based and uncertainty-based ($U_{\mathrm{cert}.}/U_{\mathrm{uncert}.}$) active learning methods on two clinical and four public health reviews. The average utility is computed when 5%, 10%, 25% and 100% of the instances are manually labelled. Moreover, the last two rows of the table record the average gain in utility that is achieved by the two semi-supervised models across all systematic review datasets. In terms of average utility, the semi-supervised approach significantly outperformed the baseline AL method (across the n=6 datasets with a one-tailed sign test with p=0.0156 and a significance level of 0.05) for certainty-based sampling at 10%, 25%, and 100% of citations manually labelled and at 25% and 100% manual labelling for uncertainty-based sampling.

**Table 2:** Average utility performance (%) of certainty-based and uncertainty-based active learning models ($U_{\mathrm{cert}.}/U_{\mathrm{uncert}.}$) when a seed size of 5%, 10%, 25% and 100% of the instances are used for training across two clinical (i.e., COPD and Proton Beam) and four public health reviews (i.e., Cooking Skills, Sanitation, Tobacco Packaging and Youth Development). Emboldened values indicate the highest utility performance for a given seed size and dataset. The table also summarises the average standard deviation (i.e., average SD) of utility values across 10 runs while the last two rows of the table report the average gain in utility over the baseline AL method that is achieved by the two semi-supervised methods, namely SemiBow and SemiSpectral, across all six systematic review datasets. The superscript ★ indicates that the corresponding semi-supervised method significantly outperformed the AL method (across the n=6 datasets with a one-tailed sign test with p=0.0156 at a level of 0.05).

Dataset	Method	Percentage of citations manually screened				
		5%	10%	25%	100%	
		$U_{\mathrm{cert}.}/U_{\mathrm{uncert}.}$	$U_{\mathrm{cert}.}/U_{\mathrm{uncert}.}$	$U_{\mathrm{cert}.}/U_{\mathrm{uncert}.}$	$U_{\mathrm{cert}.}/U_{\mathrm{uncert}.}$	
						
	AL	60.92/73.71	64.37/80.63	78.88/90.10	92.35/95.14	
COPD	SemiBow	65.33/77.26	75.41/80.57	86.19/89.36	94.19/94.91	
	SemiSpectral	65.30/**88.71**	74.35/**86.87**	85.65/**91.93**	94.06/**95.55**	
						
	AL	47.57/79.23	62.57/88.31	82.65/94.39	93.33/96.21	
Proton	SemiBow	50.68/**79.61**	68.34/88.90	84.94/94.72	93.84/96.31	
Beam	SemiSpectral	53.65/79.57	70.49/**89.08**	86.03/**94.87**	94.12/**96.37**	
						
	AL	46.66/47.59	59.40/60.56	75.17/73.69	89.68/88.59	
Cooking	SemiBow	56.26/57.13	68.05/66.11	80.75/76.77	91.43/89.07	
Skills	SemiSpectral	**60.71**/53.17	**70.65**/64.98	**81.96**/76.24	**91.78**/88.98	
						
	AL	24.44/**25.61**	32.10/32.23	52.09/48.49	82.49/80.63	
Sanitation	SemiBow	24.27/24.68	35.37/32.82	54.54/48.36	83.18/80.59	
	SemiSpectral	24.37/17.30	**37.71**/31.52	**57.38**/54.03	**83.95**/82.09	
						
	AL	45.70/43.48	53.96/55.79	75.35/72.70	90.85/90.06	
Tobacco	SemiBow	50.27/55.61	61.92/62.79	78.66/75.78	91.68/90.56	
Pack.	SemiSpectral	54.70/**60.78**	63.98/**68.27**	**79.24**/78.75	**91.84**/91.42	
						
	AL	22.71/28.09	31.34/36.52	51.97/56.34	82.81/83.66	
Youth	SemiBow	32.61/41.43	42.62/46.48	61.77/60.02	85.69/84.43	
Dev.	SemiSpectral	36.40/**49.91**	44.13/**53.73**	62.29/**64.56**	**85.94**/85.83	
						
	AL	5.35/3.78	2.95/2.24	1.32/0.98	0.30/0.26	
Average SD	SemiBow	8.45/4.05	4.46/2.21	1.89/1.15	0.49/0.31	
	SemiSpectral	6.95/4.11	3.54/2.48	1.49/1.18	0.39/0.35	
						
Average gain	SemiBow	5.23/6.33	7.99^★^/3.93	5.12^★^/1.55^★^	1.42^★^/0.26^★^	
over AL	SemiSpectral	7.85/8.62	9.59^★^/6.73	6.07^★^/4.11^★^	1.70^★^/0.99^★^	

Overall, it can be observed that the SemiSpectral model, using either certainty-based or uncertainty-based active learning, achieved the best utility in most cases. Regarding certainty-based active learning, the SemiSpectral model achieved performance gains over the baseline AL method ranging between 0% and 14% when 5% of the instances are used for training (an exception to this is the Sanitation review where performance improvements were observed after 10% of the instances were added to the training set), 6–14% when using 10% of the instances for training, 4–11% for a training set consisting of 25% of the instances and 1–2% after manually screening 100% of the instances. The certainty-based SemiBow model also improved upon the utility of the baseline method, although smaller improvements were observed here (i.e., ∼0–10%, 3–11%, 2–10% and 0–2% for a training set of 5%, 10%, 25% and 100% of the instances, respectively).

When comparing the utility of the uncertainty-based active learning models, we note that the SemiSpectral method demonstrated an increase over the performance of the baseline approach in four out of the six reviews (i.e., COPD, Cooking Skills, Tobacco Packaging and Youth Development) when using between 5% and 25% of the instances for training. The uncertainty-based SemiBoW model outperformed the baseline approach in three systematic review datasets (Cooking Skills, Tobacco Packaging and Youth Development) for a training set size of 5–25% of the instances. With regard to the clinical Proton Beam review, the semi-supervised methods obtained approximately the same utility performance as the baseline model, while in the public health Sanitation review, a higher utility (over the baseline method) is observed only after 25% of the instances was used for training.

## 4 Discussion

The results showed that our novel method demonstrates a substantial improvement in utility over both certainty [8] and uncertainty-based [2] active learning when a small sample of manually screened citations was used for training. Thus, our method is able to alleviate the problem of insufficient labelled data during the initial active learning iterations. In practice, this means that the semi-supervised active learning approach is able to discover a large number of relevant citations earlier than conventional active learning methods.

O’Mara-Eves et al. [5] highlighted that the identification of eligible citations during the early stages of the screening process has a number of advantages. Firstly, human reviewers gain a better understanding of the inclusion criteria, which enables them to screen at a higher rate once they have analysed the bulk of important citations. Secondly, in screening tasks that involve screening thousands of citations, it is only necessary for the reviewing team to double-screen the initial, key citations. Afterwards, the first reviewer can screen out the remaining citations that are prioritised lower in the list (i.e., citations that are likely to be ineligible) while the second reviewer can undertake the next task of the review. Thirdly, Cohen et al. [13] suggested that the early prioritisation of eligible citations can be useful when time and cost constraints prevent reviewers from screening the complete citation list. This ensures that a high proportion of eligible citations will be screened and subsequently included in the final review.

Whilst early prioritisation of eligible citations can significantly reduce the time and resources required to develop a systematic review, existing automatic screening models, including our proposed semi-supervised method, have only been evaluated against completed systematic reviews. This retrospective evaluation protocol assumes that human reviewers screen at a constant rate, which is not always the case in actual systematic reviews. For example, reviewers tend to make cautious and slower screening decisions during the initial stage of the process, while their screening rate is expected to increase after processing a significant number of relevant citations. Based upon this, we plan to integrate the semi-supervised active learning method with ‘live’ systematic reviews and assess workload savings achieved by our method in real scenarios.

## 5 Conclusions

In this paper, we have presented a novel semi-supervised approach based on label propagation to improve the efficiency of the citation screening phase of systematic reviews. The semi-supervised method leverages both labelled and unlabelled citations to enhance the classification performance during the early cycles of active learning (when few instances have been manually annotated). In our approach, we build upon the assumption that similar instances are likely to share the same class label. Accordingly, the method propagates classification codes from known instances to nearby unlabelled instances. We explored two vector space representations: (a) bag-of-words and (b) a data-dependent, spectral embedding. The spectral embedded feature space was shown to be effective in both clinical and public health reviews and the semi-supervised method improved the performance of both certainty and uncertainty-based active learning when a limited number of citations was available for training. Thus, the method can be applied successfully in developing systematic reviews in a manner that minimises cost and maximises time efficiency.

## Conflict of interest

The authors declared that there is no conflict of interest.

## Footnotes

1 http://www.researchgate.net/project/The-Embase-project.

2 http://eppi.ioe.ac.uk/cms/.

3 Based on some preliminary experiments, we chose k=3 to achieve a reasonable increase in the number of training examples with the semi-supervised label propagation. The supplementary material contains some experiments for different values of *k*, which show that larger values of *k*, namely k=30, show larger gains in utility based on larger increases in yield, while simultaneously increasing the burden. Depending on the goals of the screening prioritisation, choosing a larger value of *k* may be appropriate for a review.
