# Supplementary material for: A semi-supervised approach using label propagation to support citation screening
Source: J Biomed Inform. 2017 Aug;72:67–76. doi: 10.1016/j.jbi.2017.06.018 (PMC5726085; doi:10.1016/j.jbi.2017.06.018)
Supplement: Supplementary data 1 [file mmc1.pdf]

Supplementary material of the manuscript: "A semi-supervised approach using label propagation to support citation screening"

Georgios Kontonatsios[a], Austin J. Brockmeier[b], Piotr Przybyła[a], John McNaught[a], Tingting Mu[a], John Y. Goulermas[b], Sophia Ananiadou[a,*]

[a]*National Centre for Text Mining, School of Computer Science, University of Manchester, Manchester, United Kingdom*
[b]*School of Electrical Engineering, Electronics and Computer Science, University of Liverpool, Liverpool, United Kingdom*

1. Selection of number of nearest neighbours for label propagation

We investigate the performance of the semi-supervised approach as a function of the number of nearest neighbours that is used to transfer classification codes from manually labelled to unlabelled instances (i.e., k parameter of the model). To this end, we incrementally increase the k parameter of a semi-supervised certainty-based model (i.e., SemiSpectral-AL-C) and we record the utility and burden performance of the model across one clinical (i.e., Proton Beam) and one public health review (i.e., Tobacco Packaging). As a baseline method, we use a certainty-based active learning model without label propagation (i.e., AL-C).

We compare the utility (Figure 1a) and burden (Figure 1b) performance of 5 active learning models, namely SemiSpectral-AL-C (for $k = \{1, 3, 10, 30\}$) and the baseline AL-C method, when applied to the Proton Beam clinical review. It can be noted that the utility performance of the SemiSpectral-AL-C method increases as the k parameter is raised above $k = 10$. The SemiSpectral-AL-C method achieves the best utility performance when using a k parameter of 30. However, the semi-supervised method (for $k = 30$) shows a substantially increased burden performance when compared to the baseline AL-C method. This indicates that a large value of k may result in an increased number of false positive predictions. For smaller values of k (e.g., $k = 3$), the SemiSpectral-AL-C method obtains relatively smaller performance gains (in terms of the utility metric) but the semi-supervised model is able to maintain a reduced burden performance.

Figures 1a and 1b illustrate the utility and burden performance, respectively, of the SemiSpectral-AL-C (for $k = \{1, 3, 10, 30\}$) and AL-C methods when applied to the the Tobacco Packaging public health review. Similarly to the clinical review, the SemiSpectral-AL-C obtains a superior utility performance for $k = 30$ on the public health review but with a considerably increased screening burden when compared to the baseline method.

The results demonstrate that the choice of k has a large impact on utility in the early screening stages for a certainty-based model, and depending on the goals of the screening prioritisation, selecting a higher k may be appropriate.

2. Performance graphs

We provide the evaluation results (i.e., yield/burden and utility) of six active learning screening methods, namely: a) active learning with certainty sampling (AL-C) [1], b) active learning with uncertainty sampling (AL-U) [2], c) two semi-supervised active learning models that propagate classification labels using a bag-of-words feature space (i.e., SemiBoW-AL-C for certainty sampling and SemiBoW-AL-U for uncertainty sampling), d) two semi-supervised active learning methods that use a spectral embedded space for label propagation (SemiSpectral-AL-C and SemiSpectral-AL-U). The semi-supervised models (SemiBoW-AL and SemiSpectral-AL) are new automatic screening methods proposed in this work while the two active learning modes, namely AL-C and AL-U, were previously presented by [1] and [2], respectively, and are used in this study as baseline methods. All methods use linear SVMs.

*corresponding author
Email address: sophia.ananiadou@manchester.ac.uk (Sophia Ananiadou)

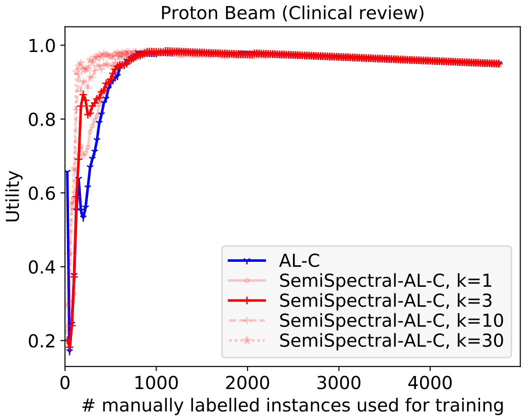

(a) Utility performance of active learning models on the Proton Beam clinical review

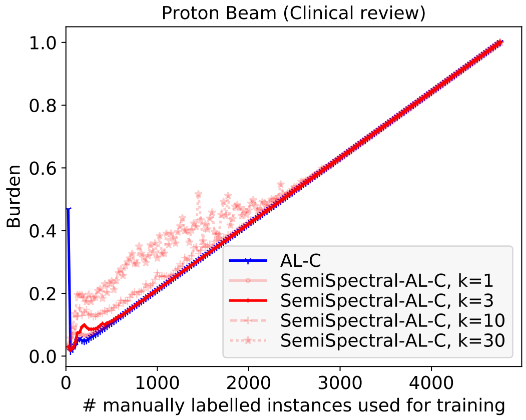

(b) Burden performance of active learning models on the Proton Beam clinical review

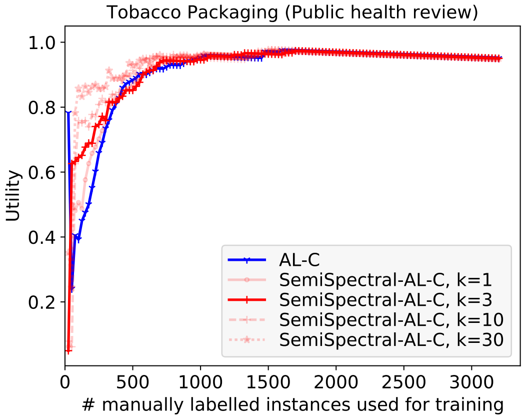

(c) Utility performance of active learning models on the Tobacco Packaging public health review

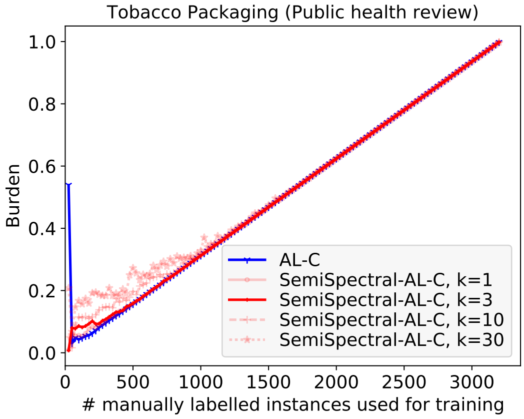

(d) Burden performance of active learning models on the Tobacco Packaging public health review

Figure 1: Utility and burden performance achieved by certainty-based active learning models (i.e., AL-C and SemiSpectral-AL-C) when applied to a clinical (Proton Beam) and a public health (Tobacco Packaging) review. The figures illustrate the performance of the SemiSpectral-AL-C model for different values of the k nearest neighbour parameter (i.e., $k = \{1, 3, 10, 30\}$). For $k = 3$, the semi-supervised model shows a substantially improved utility performance when compared to the baseline AL-C method with only a small fluctuation of the burden performance.

Figures 2–7 show the yield, burden and utility performance achieved by the automatic screening methods when applied to two clinical and four public health reviews. Regarding utility, we also record the performance of a conventional, manually conducted citation screening process (i.e., *Manual*).

3. References

[1] M. Miwa, J. Thomas, A. O'Mara-Eves, S. Ananiadou, Reducing systematic review workload through certainty-based screening, Journal of Biomedical Informatics 51 (2014) 242–253.

[2] B. C. Wallace, T. A. Trikalinos, J. Lau, C. Brodley, C. H. Schmid, Semi-automated screening of biomedical citations for systematic reviews, BMC Bioinformatics 11 (2010) 1.

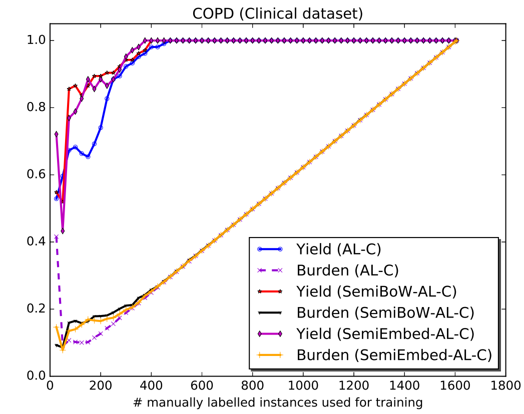

(a) Yield and burden performance of certainty-based active learners

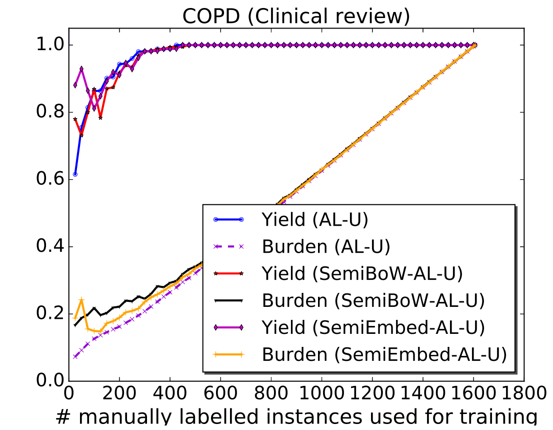

(b) Yield and burden performance of uncertainty-based active learners

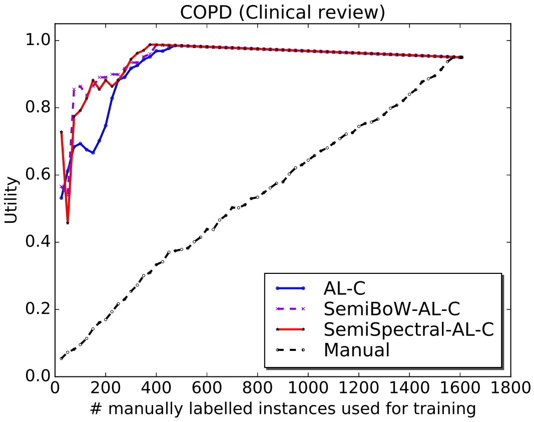

(c) Utility performance of certainty-based active learners

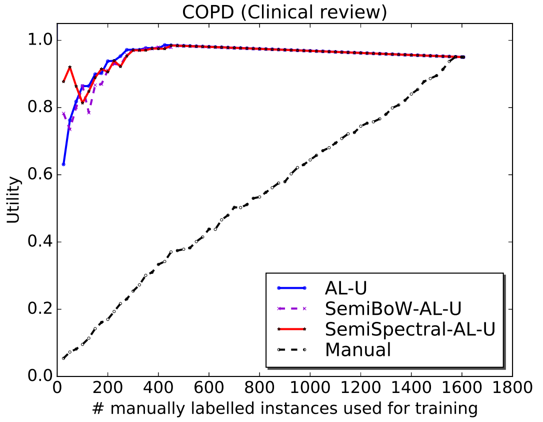

(d) Utility performance of uncertainty-based active learners

Figure 2: Yield, burden and utility performance achieved by certainty and uncertainty-based active learning models when applied to the clinical COPD review.

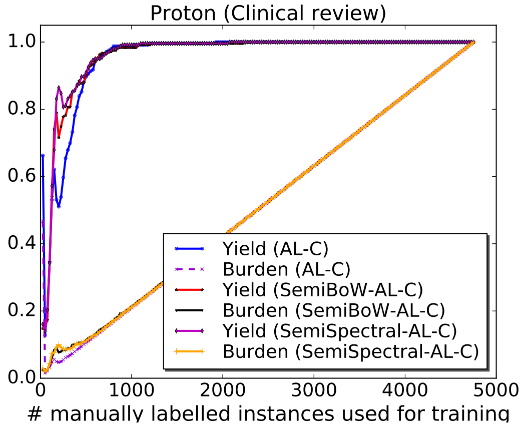

(a) Yield and burden performance of certainty-based active learners

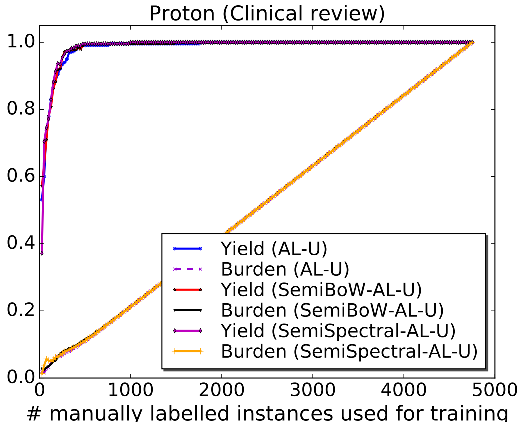

(b) Yield and burden performance of uncertainty-based active learners

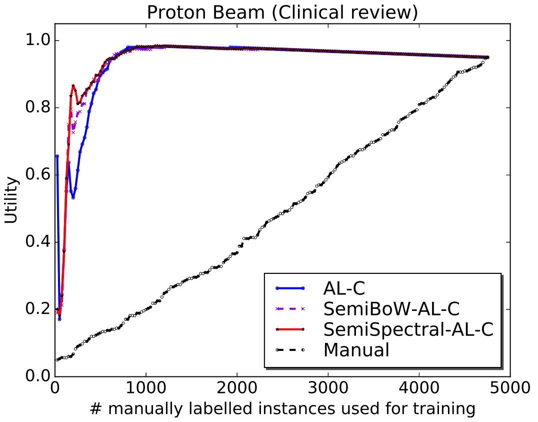

(c) Utility performance of certainty-based active learners

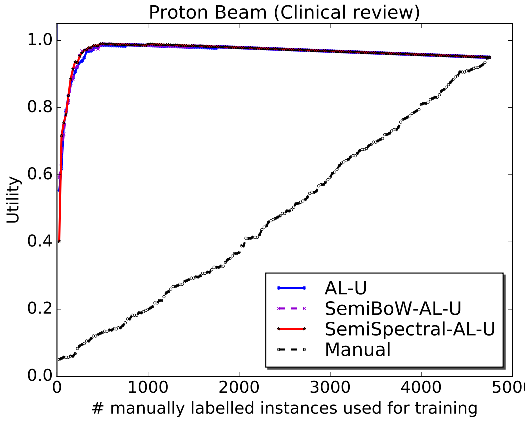

(d) Utility performance of uncertainty-based active learners

Figure 3: Yield, burden and utility performance achieved by certainty and uncertainty-based active learning models when applied to the clinical Proton Beam review.

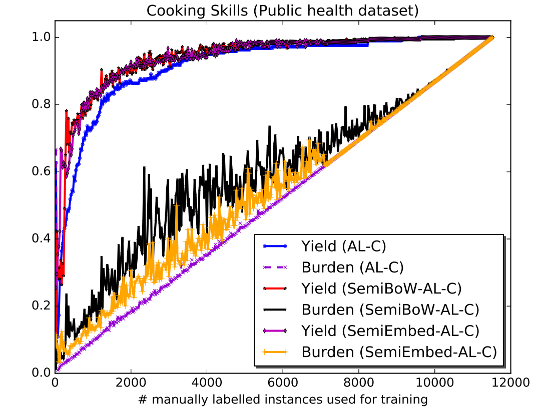

(a) Yield and burden performance of certainty-based active learners

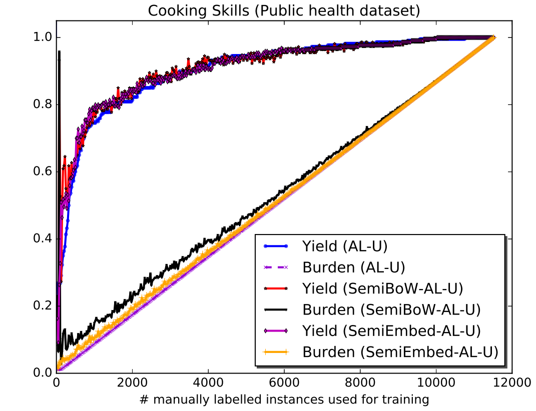

(b) Yield and burden performance of uncertainty-based active learners

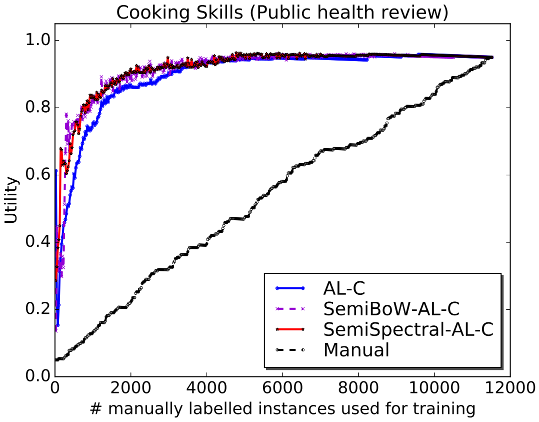

(c) Utility performance of certainty-based active learners

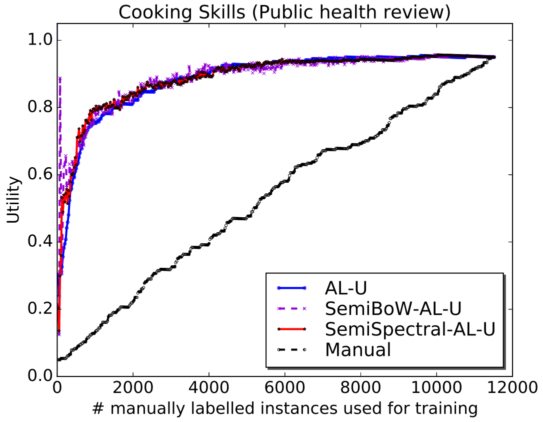

(d) Utility performance of uncertainty-based active learners

Figure 4: Yield, burden and utility performance achieved by certainty and uncertainty-based active learning models when applied to the public health Cooking Skills review.

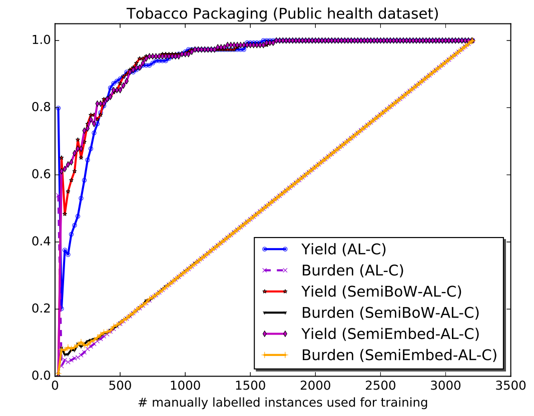

(a) Yield and burden performance of certainty-based active learners

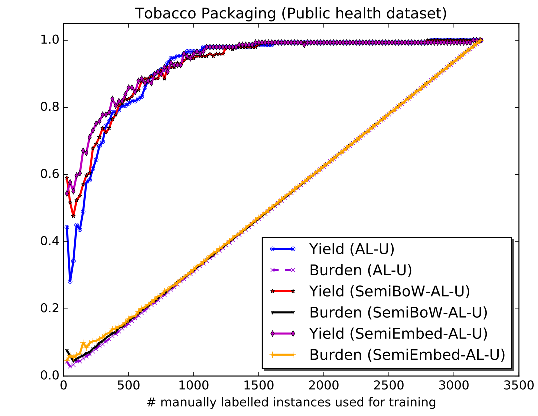

(b) Yield and burden performance of uncertainty-based active learners

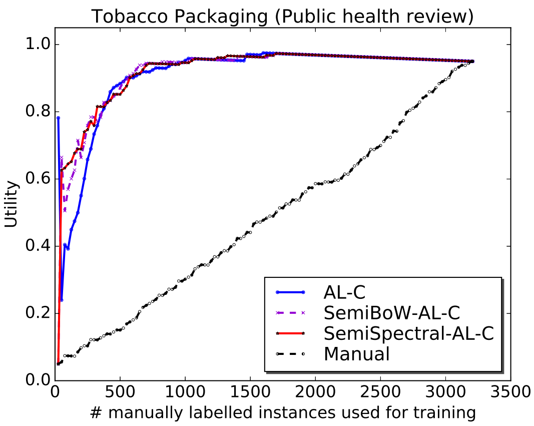

(c) Utility performance of certainty-based active learners

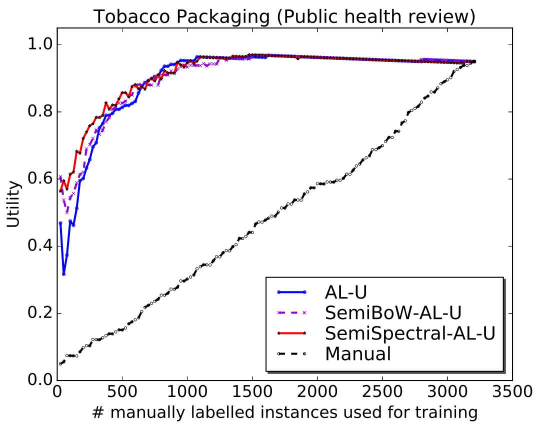

(d) Utility performance of uncertainty-based active learners

Figure 5: Yield, burden and utility performance achieved by certainty and uncertainty-based active learning models when applied to the public health Tobacco Packaging review.

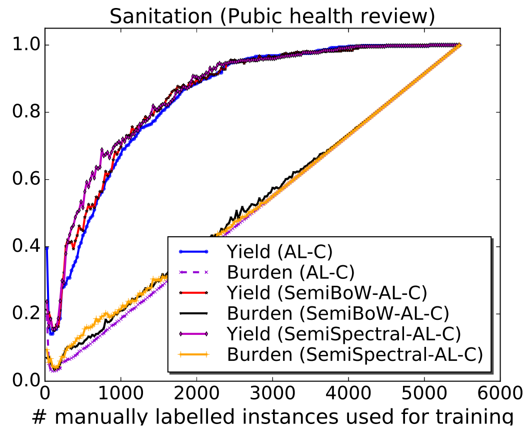

(a) Yield and burden performance of certainty-based active learners

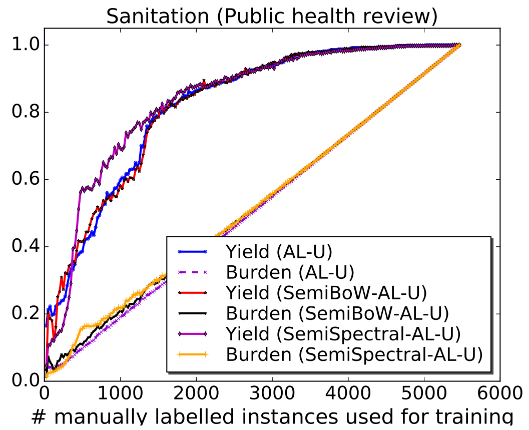

(b) Yield and burden performance of uncertainty-based active learners

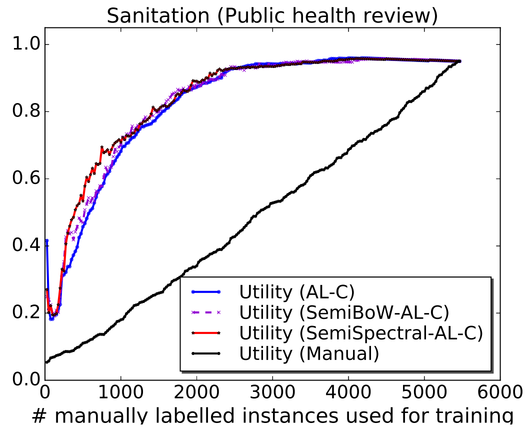

(c) Utility performance of certainty-based active learners

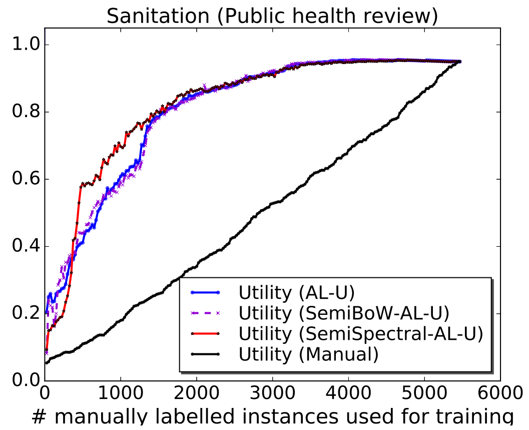

(d) Utility performance of uncertainty-based active learners

Figure 6: Yield, burden and utility performance achieved by certainty and uncertainty-based active learning models when applied to the public health Sanitation review.

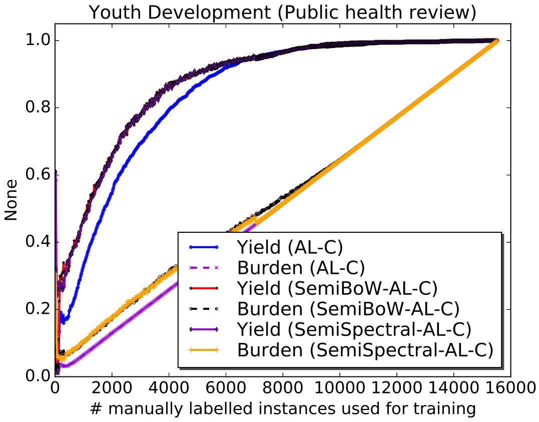

(a) Yield and burden performance of certainty-based active learners

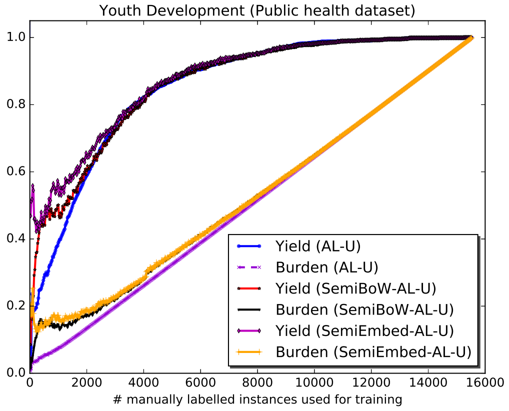

(b) Yield and burden performance of uncertainty-based active learners

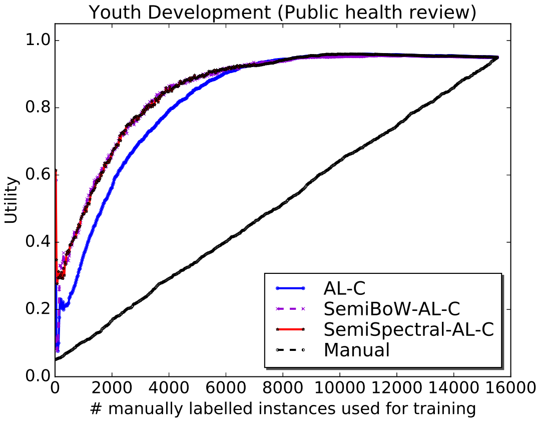

(c) Utility performance of certainty-based active learners

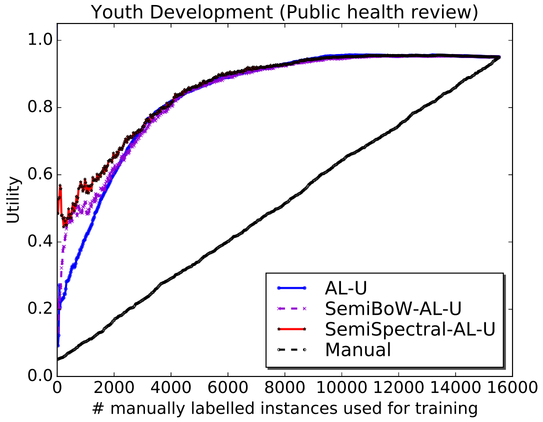

(d) Utility performance of uncertainty-based active learners

Figure 7: Yield, burden and utility performance achieved by certainty and uncertainty-based active learning models when applied to the public health Youth Development review.
